# Extraordinary high ductility/strength of the interface designed bulk W-ZrC alloy plate at relatively low temperature

**DOI:** 10.1038/srep16014

**Published:** 2015-11-04

**Authors:** Z. M. Xie, R. Liu, S. Miao, X. D. Yang, T. Zhang, X. P. Wang, Q. F. Fang, C. S. Liu, G. N. Luo, Y. Y. Lian, X. Liu

**Affiliations:** 1 Key Laboratory of Materials Physics, Institute of Solid State Physics, Chinese Academy of Sciences, Hefei 230031, China; 2 University of Science and Technology of China, Hefei 230026, China; 3 Institute of Plasma Physics, Chinese Academy of Sciences, Hefei 230031, China; 4 Southwestern Institute of Physics, Chengdu, China

## Abstract

The refractory tungsten alloys with high ductility/strength/plasticity are highly desirable for a wide range of critical applications. Here we report an interface design strategy that achieves 8.5 mm thick W-0.5 wt. %ZrC alloy plates with a flexural strength of 2.5 GPa and a strain of 3% at room temperature (RT) and ductile-to-brittle transition temperature of about 100 °C. The tensile strength is about 991 MPa at RT and 582 MPa at 500 °C, as well as total elongation is about 1.1% at RT and as large as 41% at 500 °C, respectively. In addition, the W-ZrC alloy plate can sustain 3.3 MJ/m^2^ thermal load without any cracks. This processing route offers the special coherent interfaces of grain/phase boundaries (GB/PBs) and the diminishing O impurity at GBs, which significantly strengthens GB/PBs and thereby enhances the ductility/strength/plasticity of W alloy. The design thought can be used in the future to prepare new alloys with higher ductility/strength.

Tungsten (W) is a kind of refractory metals that keeps its body-centered cubic crystal structure from room temperature to its high melting temperature of 3410 °C. W offers excellent compatibility with liquid metals, high thermal conductivity (174W/(m·k)), low sputtering yield, high stability and high hardness/strength, which all together can result in longer component lifetime, and is thus appealing for many important high temperature applications such as plasma-facing materials (PFMs) in future fusion reactors, solid target in spallation neutron source as well as critical components in rockets and missiles^1^,^2^. However, the utilization of W based materials is still limited to filaments, electrodes and heaters where W is used in a form of wire or sheet because the fracture resistance of W products is available mainly in these geometries with the optimally deformed structures achievable via heavy plastic working processes. W based bulk (thick) materials have not successfully been applied to functional and structural materials, especially in high temperature and radiation environments, because W exhibits the serious embrittlement in several regimes, i.e., low-temperature embrittlement (relatively high ductile-brittle transition temperature (DBTT) > 400 °C), recrystallization embrittlement (recrystallization temperature ~1200 °C) and radiation embrittlement^3^,^4^,^5^. Therefore the high DBTT and low recrystallization temperature of tungsten confine its application in intermediate temperature region since recrystallization with accompanying low toughness does not allow the application at very high temperatures, while, at low temperatures, existing cracks-pre-existing ones from production or cracks developed during the components’ operation might grow. Particularly, in the nuclear field, the low temperature embrittlement and radiation induced embrittlement are the main concerns^6^,^7^.

Over recent decades, many efforts have been devoted to improve the low temperature ductility of W alloys^8^,^9^,^10^. Several approaches are employed to increase ductility and fracture toughness and to decrease the DBTT. The first one is to prepare the solid solution, for example by adding rhenium (Re) as a solute element^10^. But this approach results in softening of W alloy and is costly owing to the expensive Re. Moreover, for fusion applications, Re addition has to be restricted to fulfill low activation requirements and to avoid the formation of brittle phases due to significant transmutation of W into Re^7^,^11^. The second approach is to develop nanostructured W alloys using oxide dispersion strengthening (ODS) or addition of carbide nanoparticles^12^,^13^,^14^. For W-Y materials^12^, the all Y elements are transformed into Y_2_O_3_ during mechanical alloying owing to the high amount of O in the milled powders. This is beneficial for reducing the excess O content resulting brittlement in the materials, which exhibits high strength and a promising irradiation resistance. However, they exhibit low ductility and poor RT fracture properties with DBTT still above 400 °C. Recently, some new type W or W alloys with excellent performance were developed. For example, the small sized W-1.1%TiC with ultra-fine grains fabricated by super plastic deformation exhibits very high fracture strength up to about 4.4 GPa and appreciable bending ductility at RT^14^, and the 0.1mm thick W laminate made of several layers of W foils exhibits ductility at RT^8^. However, the inferior fabrication efficiency, economy, as well as the size of the above W or W alloys are very limited to be used as PFMs or divertors^15^.

It was clearly understood that brittle fracture in W mainly occurs along grain boundaries (GBs) due to the impurities (e.g., N and O) segregation at the GBs and the weakness of GBs with random orientations^7^,^15^. In this sense any approaches modifying impurity distribution on GBs have influences on the strength and ductility. For example, minor alloying elements such as Zr can be used to purify the GBs by reacting with O to form second phase particles ZrO_2_, which would diminish the influence of O elements on GBs and strengthen the GBs^16^. In addition, ZrC also has the same ability of capturing free O elements by forming complex W-Zr-C_x_-O_y_ particles^17^. Meanwhile, the attributes of ZrC such as high melting point (~3540 °C), high thermal stability and self-adjustment capability of the lattice constant by forming a solid solution with W or non-stoichiometric ZrC_x_ could strengthen the GBs by precipitating more fine ZrC_x_ in the grain interior and segregating at the GBs. More importantly, the lattice match of *d*_(110)W _≈ *d*_(200)ZrC _≈ 0.221 nm might introduce coherent interface between W matrix and ZrC dispersoids, which will significantly increases GB /phase boundary (PB) cohesion. The strengthening effect by carbide addition was already reported in Mo bicrystals^18^ and nanostructured Mo^19^ and confirmed in nanostructured W^5^.

In this paper, trace nano-sized ZrC particles were added into W matrix by powder metallurgical process to control the interface of GBs/PBs and diminish the free O purity at GBs and successfully fabricate large size bulk W-0.5wt.%ZrC alloy plate (hereafter abbreviated as WZC, see Fig. 1a) with excellent low-temperature ductility and high strength/plasticity. As compared with the conventional oxide dispersion strengthened (ODS, like Y_2_O_3_ and La_2_O_3_) W alloys, this carbide dispersion strengthened (CDS) W-ZrC alloy alleviates the problem that dispersoids coarsened and concentrated at the grain boundaries (in ODS-W alloys) owing to the higher melting point, higher thermal stability and self-adjustment capability of the ZrC. That’s to say, W-0.5wt.%ZrC alloy builds more harmonious distribution of the dispersoids, which is effective in simultaneous high strength and extraordinary ductility. To our best knowledge, the trace ZrC dispersion strengthened W alloy has not been reported before. The mechanical property tests indicate that the bulk WZC alloy plate exhibits plasticity and bending strength of 2.5 GPa at RT, and the DBTT of about 100 °C. The ultimate tensile strength (UTS) and total elongation (TE) are about 582 MPa and 41% at 500 °C, respectively. Both the ductility and strength are significantly improved in comparison with the commercial bulk pure W or W alloy. The outstanding low-temperature ductility and high-temperature strength/plasticity can be attributed to coherent interface in grain/phase boundaries tuned by purifying and strengthening abilities of the trace ZrC.

## Extraordinary ductility/plasticity/strength

Figure 2a presents the representative stress–strain curves of the 3-points bending (3PB) tests performed at different temperatures. With testing temperature increasing from RT to 600 °C, the mechanical behavior of WZC alloy plate changes greatly. At RT, the material exhibits obvious plasticity with a flexural strain of 3% and the fracture stress up to 2.5 GPa, which is much higher than those of the hot rolled and HIPed pure W^20^, and close to those of the small sized W–TiC alloy manufactured by a severe plastic deformation^15^,^21^. For the specimen tested at 100 °C, the strain increases to 5.0%. Since maximum bending angle of the testing machine used in the present experiments is limited (about 50^o^), a temperature at which the sample undergoes a minimum of 5.0% strain without failure can be defined as DBTT^20^,^22^. In this sense, the DBTT of WZC is about 100 °C (intuitively seen in Fig. 2b and Table 1), which is much lower than the reported value in bulk pure W^7^,^20^ and W alloys^13^. With increasing testing temperature from 100 °C to 600 °C, a clear transition from ductile to brittle regime was observed, as characterized by the decrease in yield strength defined as the stress at 0.2% plastic strain, which is listed in Table 2 and clearly demonstrated in Fig. 2c. The superior comprehensive properties of both the high strength and low DBTT set the present bulk WZC alloy with large size apart from all the previously reported pure W or W alloys, which have either low strength or high DBTT (that’s to say, poor toughness at low temperature), including hot/cold rolled and HIPed pure W^20^, ODS W^13^ and W-Re alloys^23^, as summarized in Table 3 and Fig. 2c. It was reported that the W–TiC alloys have a flexural strength of about 4.4 GPa at the corresponding flexural strain of 1.5%. In the present case however, the final flexural strengths of the WZC alloy cannot be measured because the maximum bending angle of the testing machine used in the present experiments is limited to about 50^o^, which corresponds to approximately a flexural strain of 15%. Modification of the bending set-up will be done to allow for measurement of the final flexural strengths. Therefore, the flexural yield stress was chosen to compare with the other results, as shown in Fig. 2c. It is worth noting that the yield stress of WZC is the highest among the all reported bulk W alloys in the temperature range from RT to 600 °C.

The optical images of bending tested specimens were shown in Fig. 2d. It can be seen that although the specimen exhibits plasticity at RT as small as 3%, there is already observable bending. At 100 °C, the bending angle is about 30^o^. Above 150 °C, the specimens were bent to about 50^o^ (limited by testing device) without failure, which further exhibits the excellent ductility and plasticity of bulk WZC plate. The flexural strength of 2.5 GPa and strain of 3% at RT of the present WZC plates with thickness of 8.5 mm are superior when compared with the ITER grade pure W fabricated by PLANSEE Company^24^, which exhibits weak plasticity at RT in 1.5 mm thick plate. However, as the thickness of the plate increases up to 2.0 mm, the PLANSEE W plates exhibit significant decreasing plasticity and flexural strength and finally become completely brittle at a thickness of 10 mm. The nano-indentation hardness of 6.7 GPa further confirms the extraordinary high strength of the present WZC plates, which is much higher than that of pure W and W–2Y_2_O_3_^7^,^25^.

In order to further investigate the mechanical properties, the stress–strain curves of tensile test for the samples parallel to the rolling direction are shown in Fig. 2e. It can be seen that at RT the WZC alloy plate exhibits TE and UTS of about 1.1% and 991 MPa, respectively. At 100 °C the WZC alloy plate exhibits obvious tensile plastic deformation with TE~3% and UTS ~ 1.1 GPa. When the testing temperature increases to 500 °C, UTS decreases to 583 MPa and TE increases significantly to as large as 41%, which are much higher than the previously reported values^26^.

### Mechanisms for extraordinary ductility/plasticity/strength

As well known, the mechanical properties are determined by the microstructure of materials. So it is very important to investigate the microstructure in detail. High magnification BSE-SEM analysis of WZC alloy (RD-ND) indicates that tungsten grains possess equiaxed structure (Fig. 3a) with grain sizes ranging from 0.3 to 3.5 μm and an average grain size of 1.03 μm (Fig. 3b), as a result of the dynamic recrystallization of WZC alloy during the hot-rolling process. The black contrast dots, most of which are homogeneously dispersed in the grain matrix (as indicated by red solid arrows in Fig. 3a and its size distribution is shown in Fig. 3c) and a small fraction of which segregate at the grain boundaries (as indicated by red/blue open arrows in Fig. 3a and the size distribution is presented in Fig. 3d), correspond to the second phase particles as confirmed by the later TEM analysis.

Further TEM analysis gives more detailed information on microstructure as shown in Fig. 3e–g. Fine W grains and nano-scaled second phase particles have been achieved by strictly controlling the mechanical alloying process, hot-rolling temperature and the amount of deformation. The detailed deformation processes are presented in the Methods section. During hot-rolling process, deformation and dynamic recrystallization occurred at the same time. Intuitively, the deformation introduced a certain amount of dislocations which are mainly influenced by the deformation quantity in each step of rolling. In addition, the dynamic recrystallization, which resulted from the rearrangement of dislocations and accompanied with the formation and growth of the sub-grains, occurred due to the driving force of the high temperature during hot rolling. In detail, the extensive dynamic recrystallization gave an essentially equiaxed fine grain structure which is attributed to the sequential structural evolution starting from dislocation cells to polygonized dislocation walls (PDWs, see Figs 3e,f), then to partially transformed boundaries (PTBs, see Fig. 3e) and finally to fine-grained (FG) structure. In contrast, this innovative processing route is quite different from the cold-rolled one which leads to elongated grains and obvious anisotropy and instability of the grains^25^,^27^,^28^.

For the nano-scaled particles, most of them disperse in tungsten grains interior (Fig. 3a,f). These intragranular particles can generate, pin down and thus accumulate dislocations within the grains during the deformation process, as indicated by the solid arrows in Fig. 3f. The pinned and accumulated dislocations effectively raise the strength and simultaneously improve the ductility of FG alloys, as reported in ref. 29. In addition, some particles tightly bounding to the GBs could impede GB sliding, as indicated by the open arrows in Fig. 3a,g. The size distributions of the particles shown in Fig. 3c,d indicate that the second phase particles locating in W grains have an average size of 51 nm (with total area fraction of 79%) covering a range from 29 to 200 nm, while the particles at W GBs show bimodal distribution which contains relatively small particles with average particle size of 60 nm ranging from 40 to 200 nm (as indicated by the red open arrows in Fig. 3a), and a small fraction of large particles with average particle size of 385 nm ranging from 250 to 400 nm (as indicated by the blue open arrows in Fig. 3a). It is worth to point out that the small particles at GBs are dominantly ZrC while the large particles are W-Zr-C-O complexes.

The details of the interface structure between the W matrix and second phase particles in WZC alloy were investigated with high-resolution transmission electron microscopy (HRTEM, viewed along [001]). More than 50 intragranular nanoparticles were analyzed using HRTEM and the selected area electron diffraction pattern (SAEDP). Here, as an example, the typical HRTEM lattice image and SAEDP of a spherical particle with diameter of about 50 nm are given, as shown in Fig. 4a,b, respectively. The SAEDP reveals that the particle is a face centered cubic (fcc) structure. Combined with the EDX results that the atomic ratio of W : Zr : C in the fine particle is about 2 : 52 : 50, it could be concluded that the intragranular nano-particles are fcc cubic ZrC. It should be noted that the ordered tiny diffraction spots along [200]_ZrC_ direction in the SAEDP of ZrC (Fig. 4b) are caused by super-lattice structure in ZrC (see the red square area A in Fig. 4a and the corresponding fast Fourier transformed (FFT) image in Fig. 4c). Additionally, the square area B containing the phase boundary of particle-matrix without super-lattice structure has a quite similar diffraction pattern (see its FFT image in Fig. 4d) to that of W-ZrC (see Fig. 4b) and independent cubic ZrC (see Fig. 4c) except for the missing tiny diffraction spots along [200]_ZrC_. Based on the above analysis, it is clear that the Kurdjumov–Sachs (K–S) orientation relationship exists between the ZrC dispersoid and W matrix. The K–S relationship satisfies the following: (1 1 1)fcc//(1 1 0)bcc, and [1, −1, 0]fcc//[1, −1, 1]bcc.

The more intuitive magnifying HRTEM of PB shown in Fig. 4e exhibits perfect coherent structure interface between W matrix and ZrC dispersoids.

Actually, this kind of coherent structure always exists in all the particle-matrix phase boundaries in W-ZrC combination including both intragranular (Fig. 4) and intergranular (Fig. 4f) ZrC particles in WZC alloy. In Fig. 4f, a clubbed ZrC particle locates across two tungsten grains’ (G1 represents the right one and G2 represents the left one) boundary, just like a deadbolt tightly locking the two tungsten grains, which could obstruct the grain boundary sliding and thus significantly increase the cohesion of GBs. Because of the different orientation of G1 and G2, only semi-coherent structure appears between the ZrC dispersoid and G2 if observing along the [001] direction as shown in Fig. 4g.

It is well known that the coherent or semi-coherent internal boundaries play an important role in strengthening materials^21^,^30^. The completely coherent or semi-coherent interfaces between W matrix and the intragranular or intergranular ZrC dispersoids induce the good ductility/strength of W-ZrC alloy. Intuitively, the ZrC nanoparticles in the grain can cause the dislocations pinned and accumulated, which can effectively improve the strength of the alloy. Moreover, there is a channel for the gliding of dislocations along coherent structure between particle and matrix interfaces^30^, which would help sustain work hardening and uniform elongation and thus enhance the ability to accommodate plastic deformation. As a result, the coherent structure and/or semi-coherent structure with K-S orientation not only strengthen the weak GBs in pure W and PBs but also absorb and store dislocations, which significantly increase the ductility and strength of alloys. On the other hand, if the internal boundaries introduced by addition of the unsuitable dispersed particles are incoherent in which they do not create close crystallographic registry between regions separated by the boundaries, these incoherent boundaries would become preferential sites for crack initiation during loading and have a serious effect on the ductility^21^,^31^. The coherent interfaces play an important role in the extraordinary enhancement of both ductiliy and strength at low temperatures.

For the dispersoids with an average particle size of 385 nm, the shape, composition and crystal structure are different from those of the above analyzed small particles. Almost all of the large particles locate at the GBs of tungsten, as shown in Fig. 4h. The EDX analysis reveals that these particles consist of W, Zr, C and O elements, suggesting the existence of non-stoichiometric W-Zr-C_x_-O_y_ complex with x and y ranging from 0.1 to 0.7 and 0.1 to 0.5, respectively. The possible formation mechanism of these W-Zr-C_x_-O_y_ particles may be suggested as follows. At first the Zr, decomposed from ZrC, would capture the impurity oxygen in tungsten and form ZrO_y_ because the binding energy of ZrO_y_ is higher than ZrC^32^,^33^. Then the residual carbon atoms react with the surrounding W atoms to form WC_x_ and finally the combined non-stoichiometric W-Zr-C_x_-O_y_ would form from the initial ZrC, ZrO_y_ and WC_x_ compounds. It is important to emphasize that there is no brittle W_2_C phase in the alloys owing to the precise control of ZrC content. This purifying process effectively reduces the embrittlement effect of oxygen impurity on grain boundaries, and thus enhances the low-temperature ductility/toughness of the tungsten based alloys, as illustrated in Fig. 5.

As a summary of the above analysis, concise schematic diagrams were presented in Fig. 5 to illustrate the strengthening and toughening mechanisms. This strategy contains three main points including strengthening PBs through coherent structure, strengthening GBs through semi-coherent structure, and purifying and strengthening GBs through Zr capturing impurity oxygen. As well known, the grain/phase cohesion determines the ductility of the material, because plastic deformation is only possible if the GB/PB cohesion is high enough that the dislocation glide can be activated in the grain interior before the material fails through intergranular fracture. Therefore, the synergistic strengthening effects of the above three factors lead to the excellent ductility/strengthen/plasticity of WZC alloy plate. The coherent interface structure comes from the good compatibility of ZrC phase with tungsten matrix and the adjustable lattice constant by forming a solid solution with W and non-stoichiometric ZrC_x_. This eliminates the stress concentrations on the interfaces (including PBs and GBs), alleviates the propensity for intergranular fracture, and increases the total elongation before failure. These results emphasize again that in W alloys the phase and grain boundaries should be carefully tailored to reach high strength and ductility.

The extraordinary low temperature ductility/strength and high temperature plasticity are further confirmed by high heat flux tests. As a plasma facing material, thermal shock resistance is a very important property which is determined by the ductility and strength. As well known, the general mechanism of crack formation under single pulse electron beam loading can be mainly attributed to thermal stresses induced by temperature gradients formed during the thermal shock tests because the expansion of the volume beneath the heat loaded area was constrained by the cold and rigid bulk material^34^,^35^,^36^. That is to say, the thermal shock resistance of tungsten materials is directly related to the mechanical properties-the better low temperature ductility and higher strength, the better thermal shock resistance^37^, because the strength is high enough to resist the stress induced by thermal shock to prohibit the formation of cracks; on the other hand, the good plasticity/ductility can consume the stress by large deformation to avoid the occurrence of crack. Figure 6 presents the SEM images of sample surface after attack of thermal shocks. No cracks were detected on the samples tested at 0.66 GW/m^2^ (power density) (Fig. 6a, absorbed energy density (AED) ~ 3.3 MJ/m^2^). And there is still no crack at 0.88 GW/m^2^ (Fig. 6b, AED ~ 4.4 MJ/m^2^) despite the surface melting, which should benefit from the extraordinary plasticity and high strength. However in the samples tested at 1.1 GW/m^2^ (Fig. 6c, AED ~ 5.5 MJ/m^2^), melting and cracks appear simultaneously. In this case, the wave-like stripes along with the crack illustrate the good ductility and plasticity of WZC alloy from another side.

### Concluding remarks

In this work, we have designed and successfully manufactured carbide dispersion strengthened and malleableized W alloys with coherent interfaces between W matrix and ZrC dispersoids as well as with high thermal stability W-Zr-C_x_-O_y_ phase at GBs via modulating the interface of GB/PB by trace ZrC. An unprecedented 3PB as well as tensile ductility/strength, in terms of flexural strain and total elongation to failure, has been derived from the resulting W-ZrC alloy. The synergistic effects of the completely coherent or semi-coherent interfaces between W matrix and the intragranular or intergranular ZrC dispersoids, the formation of high thermal stability W-Zr-C_x_-O_y_ at GBs as well as fine grains have induced the extraordinary ductility/strength in bulk W-ZrC alloy plate. This design thought and manufacturing route are suitable for batch production of engineering-applied W alloy and can guide to design new alloys with higher ductility/strength.

## Methods

### Materials fabrication

**Figure 1:**
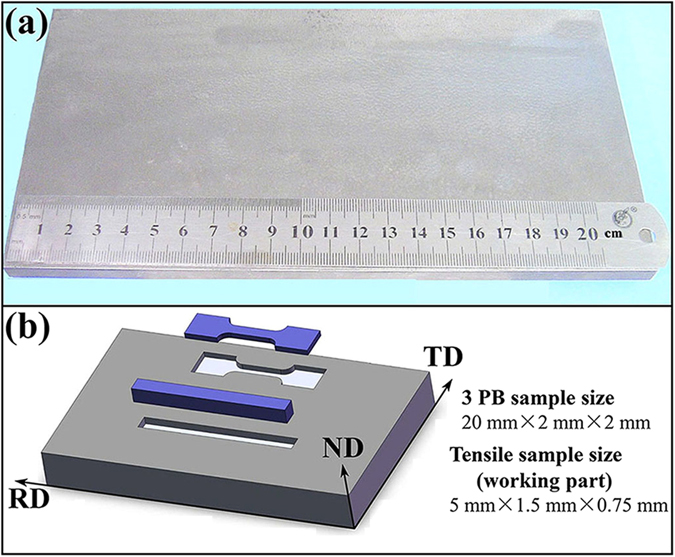
Optical image and schematic diagram of hot rolled WZC alloy plate. (**a)** Hot rolled WZC plate with thickness of 8.5 mm, width of 150 mm and length of 220 mm after cutting. (**b**) Cutting sketch of specimens from the WZC material.

**Figure 2:**
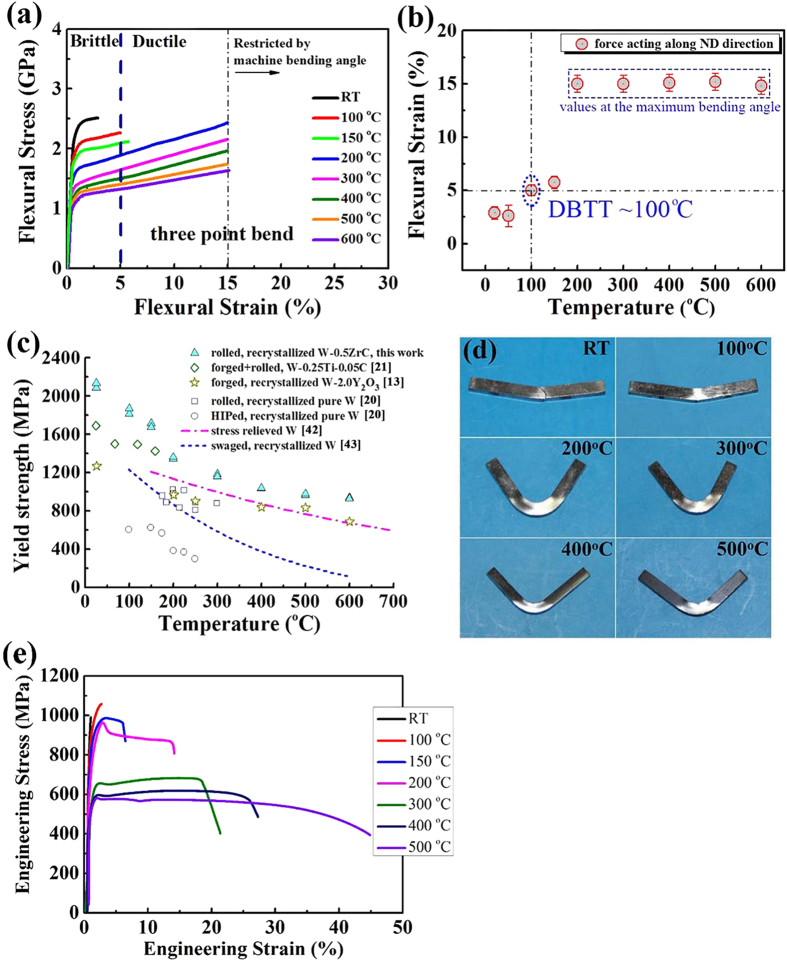
Mechanical behaviors of WZC at different temperatures. (**a**) Flexural stress-strain curves of WZC tested at different temperatures, note that values larger than a flexural strain of 15% are not accurate because of the limited bending angle of the machine. (**b)** Flexural strain of the tested samples versus temperature, DBTT is about 100 °C. (**c)** Temperature dependence of the yield strength (YS) (from 3-point bending test) of the WZC plate in comparison with available literature data. The YS of WZC is the highest among the all reported bulk W alloys. (**d)** Optical images of WZC samples after tested at different temperatures. **e**, Engineering stress-strain curves of WZC tested at different temperatures from tensile test.

**Figure 3:**
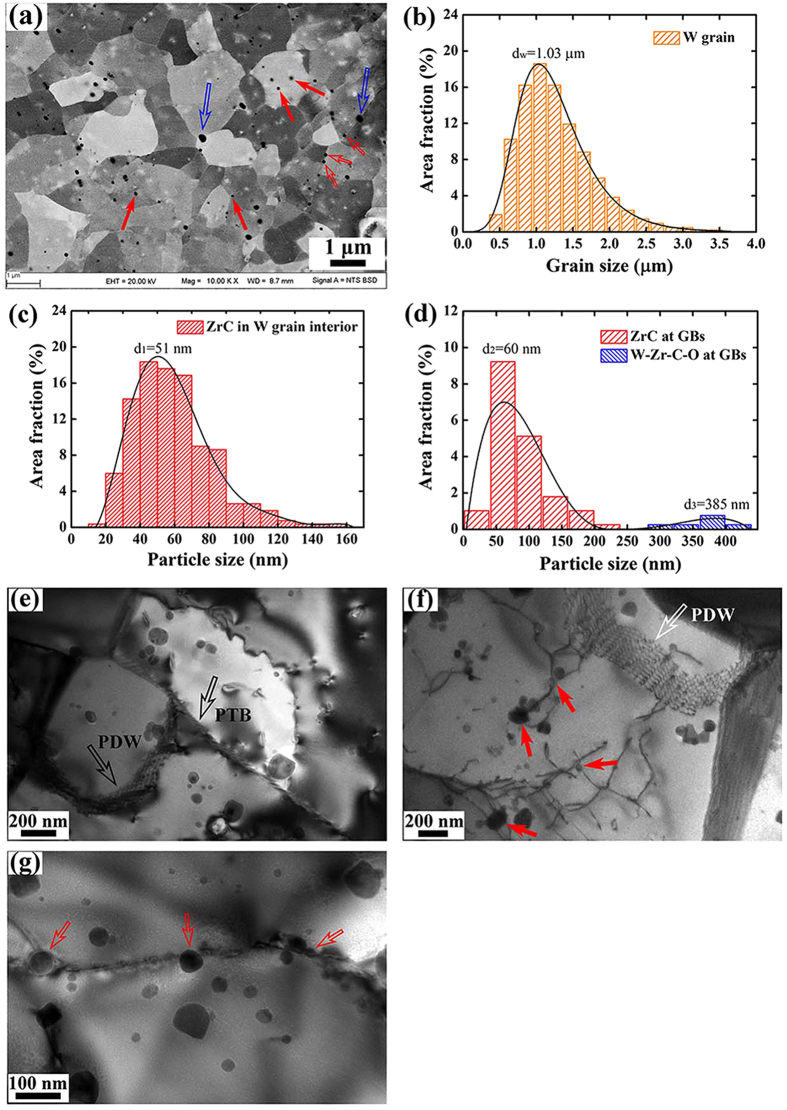
Distribution of grain/particle sizes and microstructures of WZC. (**a**) High magnification BSE-SEM image showing the tungsten grains possess equiaxed structure. The black contrast dots correspond to the second phase particles. (**b)** Grain size distribution. (**c**,**d)** ZrC and W-Zr-C_x_-O_y_ particles size distribution. (**e**) TEM images showing the formation of fine tungsten grain from PDWs transforming to PTBs during hot rolling process. (**f**) Dislocations interacting with intragranular nano particles, resulting in pinning and accumulation of dislocations inside the grain. (**g**) Some nano particles tightly bounding to the GBs could impede GB sliding. The intragranular particles (ZrC particles) are indicated by red solid arrows, and intergranular ones (ZrC/ W-Zr-C_x_-O_y_ particles) by red/blue open arrows. Note that the particles in the WZC are predominantly in the grain interior rather than at the grain boundaries.

**Figure 4:**
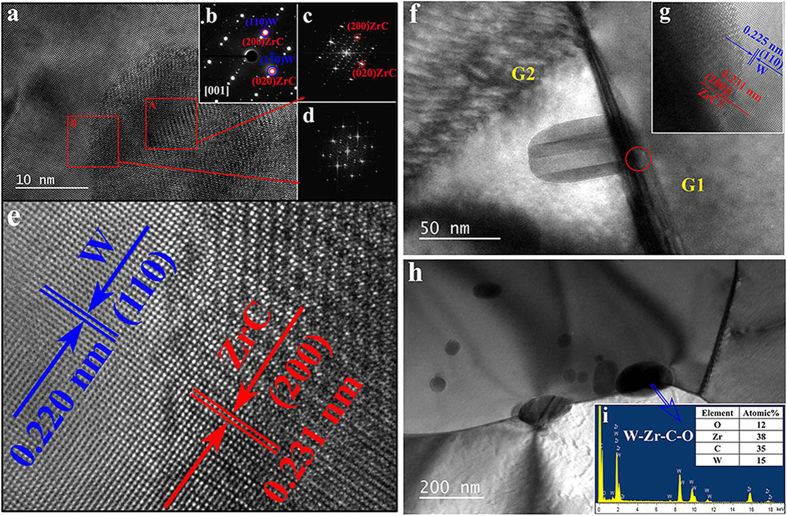
Detailed analysis of the interface structure between W matrix and second phase particles in WZC. (**a)** HRTEM image of W matrix and ZrC phase (intragranular) as viewed along [001]. (**b)** The SAEDP revealing the particle with a face centered cubic structure. **c**, Fast Fourier transform (FFT) pattern of selected red square area A on ZrC. (**d)** FFT pattern of selected red square area B at interface area between W and ZrC. It is clear that the particle-matrix phase boundaries have coherent structure like showing in high magnification (**e)**. (**f)** TEM image showing a deadbolt shaped ZrC particle (intergranular) tightly locking two tungsten grains (G1 represents the right one and G2 represents the left one). (**g**) Semi-coherent structure appears between the ZrC dispersoid and G2. (**h**) Some relative large particles locating at GBs of tungsten contain W, Zr, C and O elements through **i**, EDX analysis.

**Figure 5:**
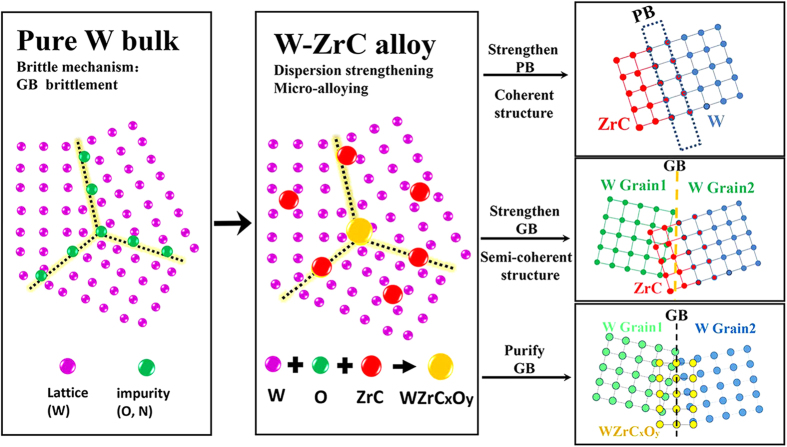
Strengthening and toughening mechanisms in WZC. This strategy contains three main points including strengthening PBs through coherent structure, strengthening GBs through semi-coherent structure, and purifying and strengthening GBs through Zr capturing impurity oxygen which leads to the excellent ductility/strengthen/plasticity of WZC alloy plate.

**Figure 6:**
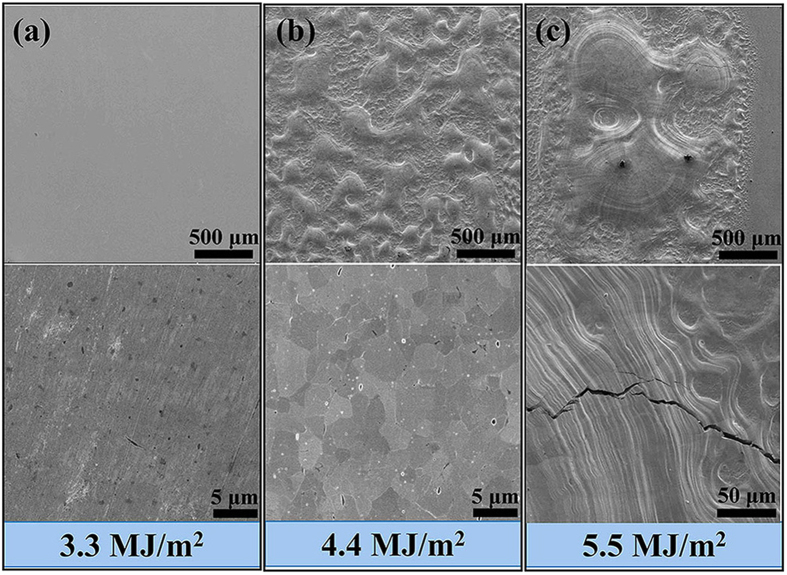
SEM images showing the thermal shock resistance properties of WZC. (**a)** No cracks were detected on the samples with absorbed energy density (AED) ~ 3.3 MJ/m^2^. (**b**) There is still no crack with AED ~ 4.4 MJ/m^2^ despite the surface melting, which should benefit from the extraordinary plasticity and high strength. (**c**), Melting and cracks appear simultaneously with AED ~ 5.5 MJ/m^2^ and the wave-like stripes along with the crack illustrate the good ductility and plasticity of WZC alloy from other side.

W–0.5 wt%ZrC alloys were fabricated using pure W powders with particle size of sub-micrometer (purity>99.9% trace metals basis), and nano-sized ZrC powders (average particle size of 50 nm, purity>99%). Powders were ball milled using high energy ball mill machine in hydrogen atmosphere for sufficient mixing. The mixed powders were subsequently sintered at 2200 °C at a pressure of 70 MPa for 20 h in vacuum. Then the sintered blank was hot-rolled into a plate with thickness of 8.5 mm (from original thickness of 24 mm, through four-step thermomechanical treatment with each step deformation of 15%, 20%, 25% and 30%, respectively, at 1650 °C), width of 150 mm and length of 220 mm (Fig. 1a after cutting). The consolidation and rolling were done by collaborating with Beijing Tianlong Tungsten & Molybdenum Co., Ltd.

### Mechanical properties tests

For tensile testing, the dog-bone-shaped samples with a cross–section of 1.5 × 0.75 mm^2^ and a working length of 5 mm along rolling direction (RD) were prepared. All tensile samples were tested using an Instron–5967 machine at a constant speed of 0.06 mm/min in vacuum. 3PB tests were performed at a constant cross-head travel speed of 0.3 mm/min on smooth (without notch) samples of (20^l ^× 2^d ^× 2^w^) mm with span width of 18 mm along with TD (Fig. 1b). Test was stopped after sample failure or after reaching a certain deflection value. For brittle materials having a liner stress–strain relation, the fracture stress (flexural strength) can be determined from the fracture stress in bending according to a linear elastic beam analysis as done in ref. 38. For both tensile and 3PB tests, three to five samples were tested at each temperature to ensure repeatability.

The hardness was measured at room-temperature using an Aglient G200 Nano-indenter (Berkovich, Diamond) with a testing depth of 2000 nm and a dwell time of 10 s.

### Microstructure characterization

TEM samples were prepared by twin-jet in Tenuple-5. The microstructure was characterized by F-Transmission electron microscope (TEM, JEM–2000FX) and high-resolution transmission electron microscopy images were recorded using JEM-ARM-200F (electron microscope operated at 200 kV, resolution transmission image 0.19 nm and lattice image 0.11 nm). The metallography of the samples was obtained using a Field Emission Gun with Scanning Electron Microscopy (FEG-SEM, ZEISS) after electrolytic polishing (in 5% sodium hydroxide aqueous solution at room temperature, 11 V and a current density of 3 mA/mm^2^). The particle/grain size and volume fraction of both intergranular (and intragranular) particles and tungsten grains have been statistically analyzed using quantitative metallography based on about 500 intergranular (and intragranular) particles and about 1000 tungsten grains in WZC alloy. The fracture surfaces of sample were characterized by field-emission scanning electron microscope (FE-SEM Sirion200, FEI).

### High heat flux tests

High heat flux tests were performed along with rolling direction using the electron beam device EMS–60 (60 kW Electron-beam Material-test Scenario) at Southwestern Institute of Physics, China, with a beam diameter of approximately 1 mm. Electrons were generated at a tungsten cathode and accelerated to a voltage of 120 kV. A homogeneous heat load distribution in the 4 × 4 mm^2^ beam spot was achieved by fast scanning (37 kHz in x-direction and 27 kHz in y-direction) of the electron beam with pulse duration of 5 ms. The electron absorption coefficient of 0.55 was determined from the ratio between the absorbed and the incident current taking into account the secondary electrons emitted from the surface.

## Additional Information

**How to cite this article**: Xie, Z. M. *et al.* Extraordinary high ductility/strength of the interface designed bulk W-ZrC alloy plate at relatively low temperature. *Sci. Rep.* **5**, 16014; doi: 10.1038/srep16014 (2015).

## Figures and Tables

**Table 1:** Summary of room-temperature properties of hot rolled WZC alloy.

Alloy	Density (g/cm^3^)	Relative density (%)	Average grain size (μm)	Hardness (GPa)	DBTT (^o^C)	Thermal conductivity (W/m·K)
W-0.5% ZrC	19.07 ± 0.04	99.7 ± 0.2	1.03 ± 0.26	6.7 ± 0.2	~100	155 ± 5

**Table 2:** Tensile and three-point bending properties of rolled WZC alloy at various temperatures

Properties	RT	100 ^°^C	150 ^°^C	200 ^°^C	300 ^°^C	400 ^°^C	500 ^°^C
Ultimate tensile strength (MPa)	991	1058	986	963	683	619	582
Elongation to failure (%)	1.1 ± 0.4	2.7 ± 1.0	6.5 ± 1.3	14.2 ± 4.2	21.3 ± 2.8	27.2 ± 3.2	41 ± 5.0
Bending strength (MPa)	2514	2266	2114	2436*	2156*	1965*	1743*
Yield strength (MPa)	2086	1815	1717	1344	1184	1035	968

^*^Value at 15% flexural strain

**Table 3:** Comparison between the present and previous reported data related to the DBTT of tungsten-based materials.

Material/Size	Working process	DBTT (K)	Dimension (mm)	Method	Ref.
W–0.5ZrC (8.5 mm thick plate)	Rolling	373	2 × 2 × 20	3PB	our
W–2Y_2_O_3_ (2 mm thick, *Φ* 95 mm)	Hot Forging	473	2 × 2 × 25	3PB	13
Pure W(10 mm thick plate)	Rolling	473	2 × 4 × 20	3PB	20
Pure W (4 mm thick)	HIP	473	2 × 3.3 × 20	3PB	20
W–0.2TiC (1 mm thick)	Forging+Rolling	440	1 × 1 × 20	3PB	21
W–0.25Ti–0.05C (1 mm thick plate)	Rolling	260	1 × 1 × 20	3PB	21
W–1%Y_2_O_3_	Injection molding	1273	3 × 4 × 27	Charpy	29
Pure W	Injection molding	1173	3 × 4 × 27	Charpy	29
W–0.5TiC	HIP + Forging	484	1 × 1 × 20	3PB	39
WL10	Swaging + Rolling	973	10 × 10 × 55	Charpy	40
Pure W (0.1 mm thick foil)	Rolling + Joinning	373	4 × 15 × 33	Charpy	41
